# A User-Friendly Protocol for Mandibular Segmentation of CBCT Images for Superimposition and Internal Structure Analysis

**DOI:** 10.3390/jcm10010127

**Published:** 2021-01-01

**Authors:** Chenshuang Li, Leanne Lin, Zhong Zheng, Chun-Hsi Chung

**Affiliations:** 1 Department of Orthodontics, School of Dental Medicine, University of Pennsylvania, Philadelphia, PA 19104, USA; llin21@upenn.edu; 2 Division of Growth and Development, Section of Orthodontics, School of Dentistry, University of California, Los Angeles, CA 90095, USA; leozz95@ad.ucla.edu; 3 Department of Surgery, David Geffen School of Medicine, University of California, Los Angeles, CA 90095, USA

**Keywords:** CBCT, segmentation, mandible, superimposition

## Abstract

Background: Since cone-beam computed tomography (CBCT) technology has been widely adopted in orthodontics, multiple attempts have been made to devise techniques for mandibular segmentation and 3D superimposition. Unfortunately, as the software utilized in these methods are not specifically designed for orthodontics, complex procedures are often necessary to analyze each case. Thus, this study aimed to establish an orthodontist-friendly protocol for segmenting the mandible from CBCT images that maintains access to the internal anatomic structures. Methods: The “sculpting tool” in the Dolphin 3D Imaging software was used for segmentation. The segmented mandible images were saved as STL files for volume matching in the 3D Slicer to validate the repeatability of the current protocol and were exported as DICOM files for internal structure analysis and voxel-based superimposition. Results: The mandibles of all tested CBCT datasets were successfully segmented. The volume matching analysis showed high consistency between two independent segmentations for each mandible. The intraclass correlation coefficient (ICC) analysis on 20 additional CBCT mandibular segmentations further demonstrated the high consistency of the current protocol. Moreover, all of the anatomical structures for superimposition identified by the American Board of Orthodontics were found in the voxel-based superimposition, demonstrating the ability to conduct precise internal structure analyses with the segmented images. Conclusion: An efficient and precise protocol to segment the mandible while retaining access to the internal structures was developed on the basis of CBCT images.

## 1. Introduction

Two-dimensional (2D) radiographs have been widely used in the field of orthodontics since 1922 [1]. At that time, when cephalometric tracing norms were established, cone-beam computed tomography (CBCT) was not available. Application of three-dimensional (3D) CBCT was first reported in 1994 [2]. As it significantly reduced radiation exposure and costs, 3D CBCT has since been broadly adopted in orthodontics after 2007 [2]. As early as 2010, Nalçaci et al. demonstrated that 3D cephalometric approaches are fairly reliable and comparable with traditional 2D cephalometry [1]. In addition, a growing body of evidence continues to demonstrate the application of CBCT in orthodontics as a front-line technology development topic [2,3,4,5,6,7]. However, the radiation exposure of cranial CBCTs is still not acceptable in most orthodontic patients, even though the dosage is dramatically reduced compared to when CBCT technology was first applied to orthodontics. To address this issue, Farronato et al. established a reliable sagittal skeletal classification system using CBCT images with a reduced field of view [8]. Overall, both clinicians and researchers are dedicating great efforts to maximize the benefits of 3D CBCT in patient care while minimizing the radiation exposure to patients.

As the only bone that connects to the cranium by unique temporomandibular joints and exhibits variable growth and remodeling responses to orthodontic treatment, the mandible has been one of the classic and central topics for evaluating patients’ growth and development and assessing the influence of orthodontic and orthopedic treatments. However, the most commonly cited method of investigating the mandible today is still 2D cephalometric radiograph-based superimposition [9]. There is no doubt that 2D image-projected 3D structure analysis not only limits the regions that can be evaluated (e.g., mandibular condyle [10], glenoid fossa [11], coronoid process, mandibular canal [12]), but also exposes the findings to inherent and unavoidable errors, such as those rooted in differences in magnification, head position, landmark identification, tracing, and reference lines and planes used for superimposition [13]. Thus, reliable 3D mandibular structure analysis technologies are in high demand, especially those that can provide valuable information previously inaccessible using 2D methods [2,3]. In particular, Kadiogly et al. stated, “Questions that were answered previously in 2D study are being asked again, and new studies are reassessing older and possibly outdated concepts with the aid of CBCT” [3].

Indeed, multiple attempts at mandibular segmentation and superimposition based on 3D CBCT imaging have been reported [13,14,15,16,17]. For instance, a significant amount of research into mandibular measurements has been conducted with such methods in the past few months [17,18,19,20], evidencing the high demand for 3D-based mandibular measurements. However, all of these pioneering methods utilize either open-source software such as ITK-Snap (www.itksnap.org) and 3D Slicer (www.slicer.org) or licensure-required software such as Mimics (Materialise, NV) [13,14,15,16,17,18,19,20]. Since the software packages mentioned above are not specifically designed for orthodontic applications, multiple software packages are generally necessary to analyze a single case. This issue significantly increases the complexity of usage and financial input, as well as time and labor costs for the clinicians or orthodontic researchers for mandible-related evaluations and investigations.

To overcome the technical, time-consuming, and financial challenges thus far associated with evaluations of the mandible, it is necessary to establish an efficient and precise mandibular segmentation protocol based on 3D CBCT that can (1) be performed with commonly used orthodontic imaging analysis software, (2) be easy to follow (not technique-sensitive), (3) access the internal structures, and (4) allow novel 3D and traditional 2D superimpositions.

## 2. Experimental Section

CBCT scans for this study were derived from preexisting clinical databases and approved by the University of Pennsylvania Institutional Review Board (IRB protocol #843611). No additional CBCT images were taken for the current study. Five CBCT scans from 3 patients were selected on the basis of the following criteria:

Patient #1: An adult female patient without craniofacial syndromes who underwent orthodontic treatment. The initial (CBCT #1, when the patient was 23 years old) and final (CBCT #2, when the patient was 25 years old) records were selected to

(1) establish the initial mandibular segmentation protocol to exclude the potential influence of gross pathologies and significant skeletal asymmetry;
(2) perform and validate the 3D voxel-based superimposition [13] with no detectable mandibular growth as determined by the American Board of Orthodontics (ABO) standard 2D landmark-based superimposition [21].

Patient #2: A late adolescent female patient diagnosed with hemifacial microsomia. The initial record (CBCT #3, when the patient was 16 years old) was selected to validate the precision and efficiency of the current protocol to segment the mandible, which presented a significant amount of asymmetry.

Patient #3: An adolescent male patient without craniofacial syndromes who underwent orthodontic treatment. The initial (CBCT #4, when the patient was 11 years old) and progress (CBCT #5, when the patient was 12 years old) records were selected to

(1) further validate the current protocol to segment the mandible;
(2) perform 3D voxel-based superimposition to determine the sensitivity of the current segmentation and superimposition protocol for detecting mandibular growth and development in the one-year interval;
(3) observe the potential growth and remodeling trends of the mandible.

**Figure 1:**
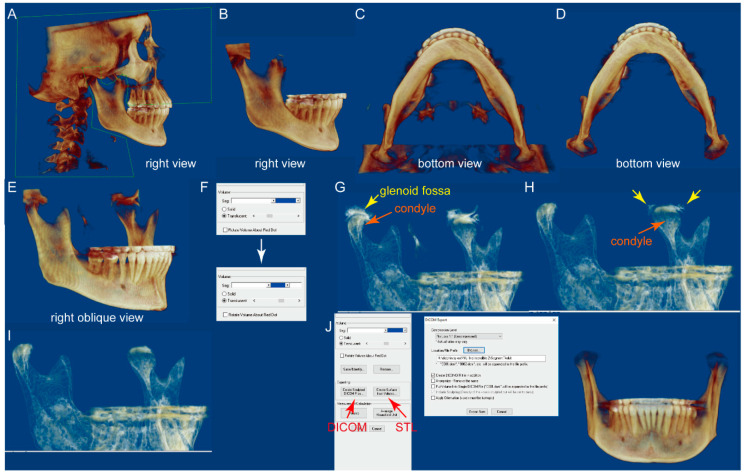
The flowchart of the mandible segmentation protocol by using the Dolphin 3D sculpting tool. (**A**,**B**) On the right view, sculpt the majority of the cranial structure and maxilla with the “free form” tool. (**C**,**D**) On the bottom view, the border of the condyles can clearly be seen. (**E**–**G**) On the right oblique view, enlarge the cone-beam computed tomography (CBCT) and change the “Seg Volumes,” which will produce a translucent view of the right condyle (orange arrow), glenoid fossa (yellow arrow), and remaining maxillary structures. (**H**) After sculpting around the left condyle in the left oblique view, return to the right oblique view. Some remaining cranial structures can be found around the left condyle (yellow arrows). Sculpt them (**I**). (**J**) The segmented mandible CBCT can either be exported as DICOM files or be used to create the surface structure and saved as an STL file.

CBCT DICOM files were all imported into the Dolphin 3D software (Dolphin Imaging; version 11.95 Premium, Chatsworth, CA; user license purchased by the University of Pennsylvania, School of Dental Medicine, Department of Orthodontics). The mandible was segmented from each CBCT with the following steps:

(1) In the “Orientation” module, set the facial midline as the midline of the full-volume CBCT and ensure that the cranial base structures and key ridges on the left and right sides overlap. This step is the same as the orientation setting for routine craniofacial CBCT assessments.
(2) In the “Sculpting tool” module, orient the CBCT to the right view, and sculpt the majority of the cranial and maxilla structures with the option “free form”, as shown in Figure 1A,B.
(3) Orient the CBCT to the bottom view in which the borders of the condyles are clearly visible. Sculpt the visible cranial and maxillary structures, as shown in Figure 1C,D.
(4) Orient the CBCT to the right oblique view (Figure 1E), enlarge the CBCT, and change the “Seg Volumes” to identify the lower and upper density values in the appropriate range to differentiate the condyle from the surrounding structures (Figure 1F). This adjustment will produce a translucent view of the right condyle (orange arrow), glenoid fossa (yellow arrow), and remaining maxillary structures (Figure 1G). Sculpt the glenoid fossa along the inferior border and remaining maxillary structures.
(5) Repeat step 4 for the left condyle with the CBCT oriented to the left oblique view.
(6) Return to the right oblique view, in which some remaining cranial structures can be found around the left condyle (Figure 1H, yellow arrows). Sculpt them (Figure 1I).
(7) Repeat step 6 for the right condyle with the CBCT oriented to the left oblique view.
(8) The segmented mandible CBCT is ready for either “Export” as DICOM files or “Create surface,” which yields an STL file (Figure 1J).

Each CBCT was segmented by the same examiner twice, with a 1-week interval in between. Each CBCT was also independently segmented by another examiner. The 3 STL files of each mandible were imported into 3D Slicer (open-source software, www.slicer.org) to evaluate intra-examiner and inter-examiner reproducibility, as well as the reliability of the current protocol using the “Model-to-Model distance” module [13]. The DICOM files of each mandible were imported into the Dolphin 3D software for voxel-based superimposition [22] using the chin and symphyseal regions [13] and internal anatomical structure analysis. To further assess the intra- and inter-examiner reliability, we used 20 additional CBCTs for mandibular segmentation by 2 individual clinicians in a blinded fashion (Figure S1). Intraclass correlation coefficient (ICC) was employed to assess the consistency of volumetric measurements on the mandibles segmented by the current protocol with IBM SPSS software (Statistical Package for Social Sciences version 26.0, Chicago, IL, USA).

## 3. Results

**Figure 2:**
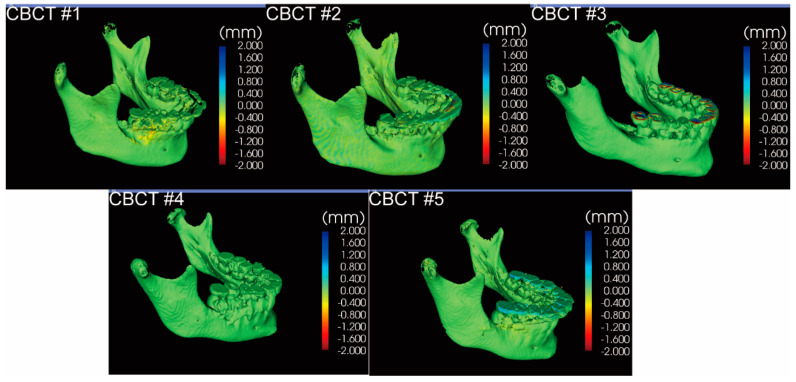
The model-to-model distance analysis in the 3D Slicer software for two segmentations performed by one examiner for each CBCT.

**Figure 3:**
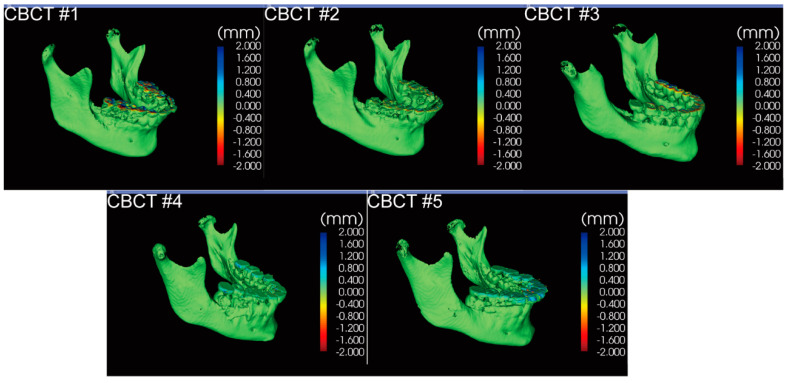
The model-to-model distance analysis in the 3D Slicer software for two segmentations individually performed by two examiners for each CBCT.

It took less than 15 min each to complete all segmentations. The volume matching results of the two segmentations from the same clinician for each CBCT are shown in Figure 2, demonstrating intra-examiner reproducibility. Volume matching was also performed with two independent segmentations from two clinicians for each CBCT, shown in Figure 3, displaying inter-examiner reproducibility. The 3D Slicer Model-to-Model distance results showed perfect surface matching of the mandibular body, ramus, and condylar regions for all five samples in Figure 2 and Figure 3, illustrating the reliability of the segmentation protocol described above. As no prominent landmarks could be found in the maxillary dentition to determine the segmentation border in this region, some volume differences were detected by 3D Slicer. Some slight surface differences were also observed in CBCT #1 around the lower right first molar region, which could be attributed to the metal crown-derived noise around this tooth. However, these negligible differences did not influence further analysis of the mandible. For the ICC analysis based on the segmentation of 20 mandibles by two clinicians, intra-examiner reliability (ICC  =  0.998; 95% CI  =  0.964–1.000) and inter-examiner reliability (ICC  =  0.998; 95% CI  =  0.908–1.000) were excellent.

**Figure 4:**
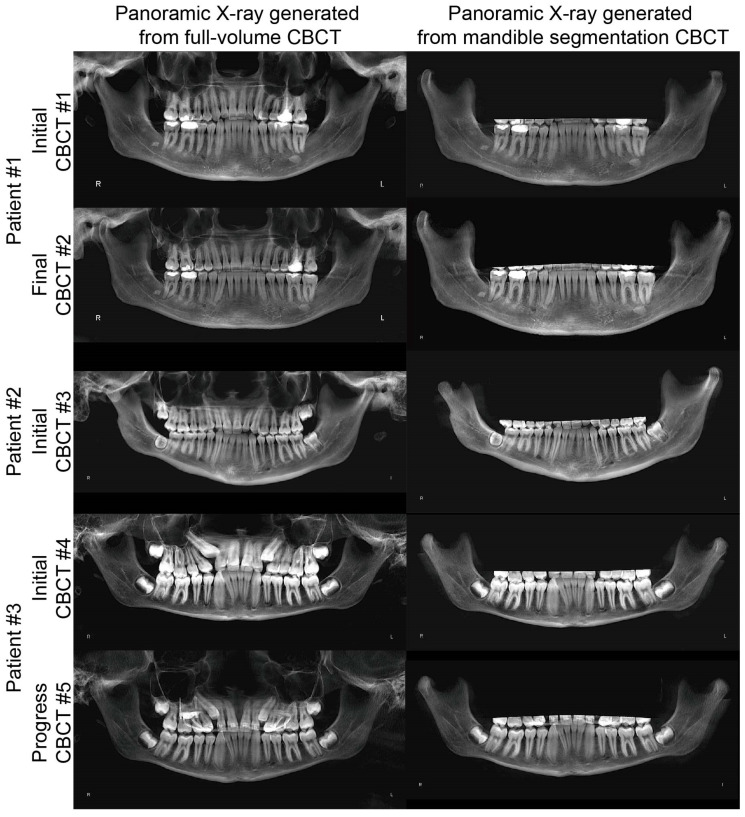
Comparison of the panoramic radiographs generated from the full-volume CBCT and those from the segmented mandible CBCT.

Comparing the panoramic radiograph images generated from the full-volume CBCT and the segmented mandible CBCT, no gross morphological differences were noted in any tested samples (Figure 4). Furthermore, the panoramic radiographs generated from the segmented mandible CBCT clearly displayed the inferior alveolar canals, tooth roots, condyles, and bone marrow (Figure 4). 

**Figure 5:**
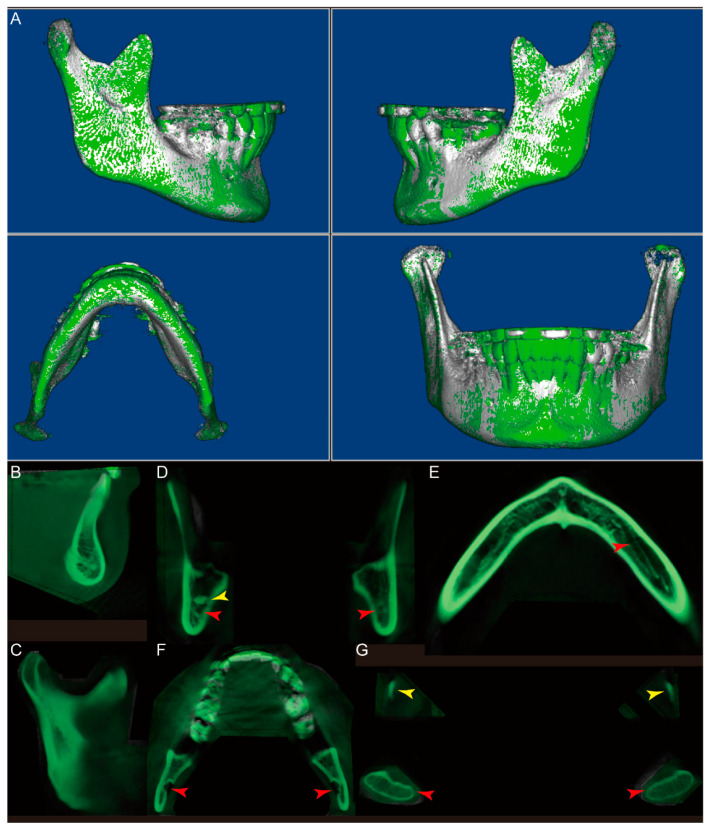
The Dolphin 3D voxel-based superimposition of the initial (white, CBCT #1) and final (green, CBCT #2) CBCT datasets of the mandible for patient #1. (**A**) The 3D reconstructed surface structure image of the mandible after superimposition. (**B**) Sagittal slice along the facial midline. (**C**) Sagittal slice in the left ramus region. (**D**) Coronal slice in the retromolar region. Yellow arrow: a radiopaque area on the right side of the mandible; red arrows: inferior alveolar canals. (**E**) Axial slice at the spinae mentalis level. Red arrow: left inferior alveolar canal. (**F**) Axial slice at the mandibular foramen level. Red arrows: mandibular foramen. (**G**) Axial slice at the level of the tips of the coronoid processes. Yellow arrows: coronoid process; red arrows: condyle.

For patient #1, the Dolphin 3D voxel-based superimposition generated evenly distributed white (initial) and green (final) interval surface colors in the mandibular body and ramus regions. In contrast, only the green color was observed in the lower anterior dentition, indicating no significant change of the mandible, while there was proclination of the mandibular anterior teeth caused by orthodontic treatment (Figure 5A). To further confirm this observation, we analyzed the superimposed mandibular CBCT images in all three planes. In the sagittal slice along the facial midline, the symphyseal regions of the initial (white) and final (green) mandibles were superimposed, while the mandibular anterior teeth were more proclined in the green than in the white segment (Figure 5B). The sagittal slice in the left ramus region displayed perfectly matched white and green images (Figure 5C). The coronal slice in the retromolar region again showed completely overlapping white and green images (Figure 5D). It is worth noting that the radiopaque area on the right side of the mandible (Figure 5D, yellow arrow) and the inferior alveolar canals on both sides (Figure 5D, red arrows) were clearly seen. In the axial slices, the left inferior alveolar canal (Figure 5E, red arrow), mandibular foramen (Figure 5F, red arrows), condyles (Figure 5G, red arrows), and tips of the coronoid processes (Figure 5G, yellow arrows) were all easily located. Furthermore, all of these slices showed complete superimposition of the initial and final images at the mandibular body and ramus regions.

**Figure 6:**
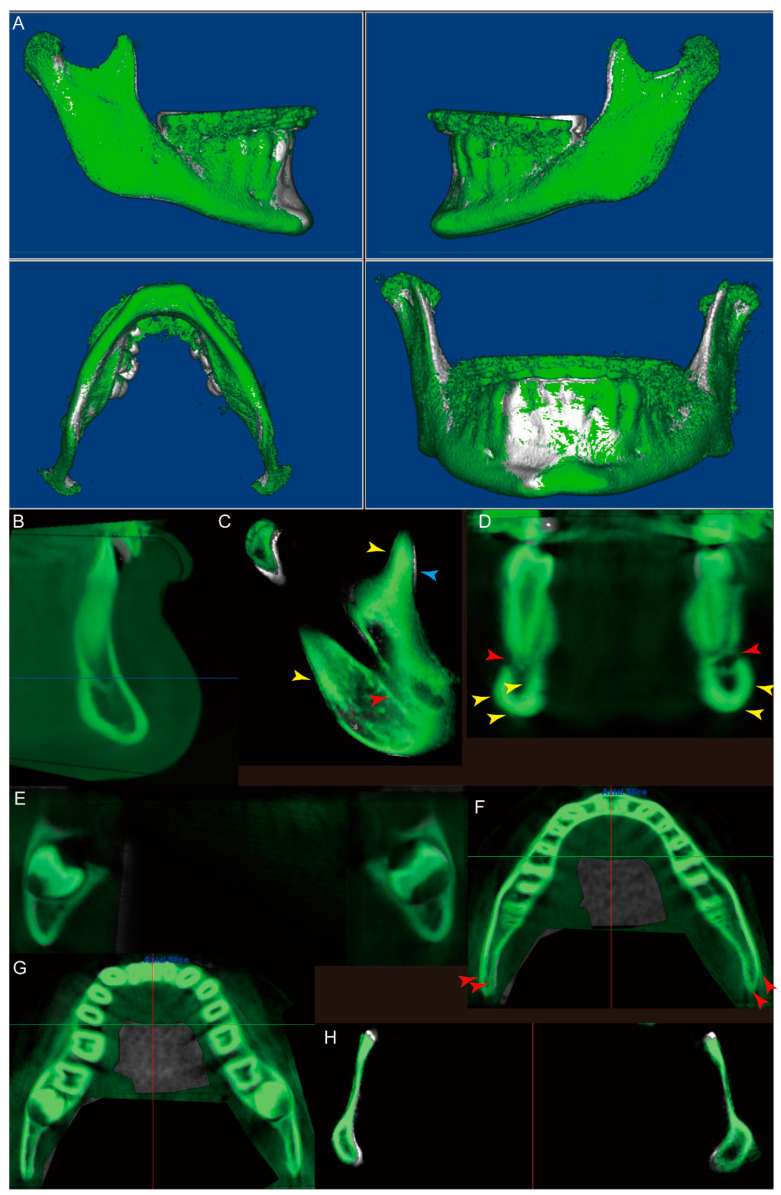
The Dolphin 3D voxel-based superimposition of the initial (white, CBCT #4) and progress (green, CBCT #3) CBCT datasets of the mandible for patient #3. (**A**) The 3D reconstructed surface structure image of the mandible after superimposition. (**B**) Sagittal slice along the facial midline. (**C**) Sagittal slice in the right ramus region. Red arrow: inferior alveolar canal; blue arrow: resorption; yellow arrows: deposition. (**D**) Coronal slice in the first premolar region. Yellow arrows: deposition; red arrows: mental foramen. (**E**) Coronal slice in the third molar region. (**F**) Axial slice at the root apex level. Red arrows: buccally moved cortical plates. (**G**) Axial slice at the cervical level of the teeth. (**H**) Axial slice at the level of the deepest portion of the mandibular notch.

Unlike the observations from patient #1, the Dolphin 3D voxel-based superimposition of patient #3 showed that the majority of the mandibular surface was green, indicating the potential growth of the mandible from the initial (white) to progress (green) time points (Figure 6A). Again, the sagittal slice along the facial midline shows superimposition of the two images at the symphyseal region (Figure 6B), while the sagittal slice in the right ramus region (Figure 6C) displays (1) superimposition of the inferior alveolar canal (red arrow), (2) resorption (blue arrow) of the anterior surface and deposition (yellow arrows) at the posterior surface of the top half of the coronoid process, (3) posterior and superior growth of the condyle, and (4) deposition (yellow arrows) at the posterior border of the ramus. For the coronal slices, in the first premolar region (Figure 6D), the mental foramen could be found and superimposed in the first premolar region (red arrows). Simultaneously, slight deposition was also noted at the buccal and inferior surfaces of the mandibular body and the inner contour of the lingual cortical plate on the right side (Figure 6D, yellow arrows). In the third molar region (Figure 6E), while the lower borders of the third molars were superimposed on both sides, deposition was found at the buccal surface of the mandibular body. For the axial slices, the slice at the root apex level (Figure 6F) showed deposition along the buccal surface of the mandibular body. In the posterior third of the mandible, both the buccal and lingual cortical plates moved buccally (Figure 6F, red arrows). The buccal movement of the buccal and lingual cortical plates in the posterior third of the mandible was also found in the axial slice at the cervical level of the teeth (Figure 6G). Finally, the axial slice at the level of the deepest portion of the mandibular notch (Figure 6H) showed (1) resorption of the anterior surface of the ramus, (2) deposition at the buccal surface of the ramus, and (3) resorption of the medial posterior surface of the condylar neck.

## 4. Discussion

For this short Communication article, our primary aim was to establish a simple protocol to segment the mandible from a full-volume craniofacial CBCT using the “Sculpting tool” provided with the Dolphin 3D Imaging software and to evaluate the precision and reliability of this protocol. In contrast to other newly established mandibular segmentation protocols, the current protocol only involves one commonly used orthodontic imaging software, which reduces the cost, time, and energy of purchasing software, multiple-software practicing, and file transfers among different software [13,14,15,16,17,22]. 

The 3D Slicer model-to-model distance analysis demonstrates perfect volume matching of the mandibular body, ramus, and condylar regions in all five tested samples. This consistency confirms the precision and reliability of the current protocol, which is not affected by notable mandibular asymmetry, such as seen in patient #2.

We also performed superimpositions using the chin and symphyseal regions [13] with Dolphin 3D voxel-based superimposition [22]. As demonstrated by the planes of patient #1, the inner contour of the cortical plate at the lower border of the symphysis, alveolar canal, and anterior and posterior border of the ramus could all be easily located. Thus, all of the anatomical structures indicated for mandibular superimposition by the ABO can be found and superimposed using this 3D voxel-based superimposition dataset. The complete superimposition of these anatomic structures confirms the precision of the 3D voxel-based superimposition method, as previously reported [13,22], and further validates the reliability of the current mandibular segmentation protocol. 

As demonstrated in the superimposition image of patient #3, we found that specifically for this patient (1) the anterior–inferior contour of the chin and the inner contour of the cortical plate at the lower border of the symphysis can be entirely superimposed for the initial and progress records, (2) there is deposition at the inferior border of the mandible at the first premolar region but not at the third molar region, and (3) the ramus demonstrates anterior border resorption and posterior border deposition. These observations align with the previously reported and accepted phenomena of mandibular growth and modeling [21]. 

Interestingly, some previously unreported growth trends were also found in this patient’s records, such as the deposition along the buccal surface of the mandible (Figure 6A,D–G) and the lateral shift of the buccal and lingual cortical plates in the posterior third of the mandible (Figure 6F,G), indicating that the mandible may not grow posteriorly along the direction of the mandibular body. There may be a contour change during growth. Furthermore, instead of a “V”-shaped growth of the condyle, only the medial posterior resorption of the condylar neck is observed in the axial slice at the most inferior level of the mandibular notch (Figure 6H). 

In fact, in the newly published article [23], by performing a longitudinal CBCT study on 25 growing skeletal class II patients, Maspero et al. found that there was significant mandibular body growth as expected, but the mandibular symphyseal angle maintained the same. This is different from what we observed in patient #3—a lateral shift of the mandible is clearly indicated in Figure 6F,G, suggesting an increasing mandibular symphyseal angle. It is worth noting that patient #3 was diagnosed as skeletal class III and had rapid maxillary expansion and facemask treatment before the progress records were taken. Thus, whether these previously unreported mandibular growth patterns resulted from a normal skeletal class III pattern of growth or from treatment requires further investigations with a larger sample size.

Using the current protocol, we could not thoroughly remove the maxillary dentition without affecting the mandibular dentition. Thus, we decided to sculpt through half of the maxillary tooth crowns. Although the remaining maxillary dentition did not appear to influence the mandible analyses, further efforts are still needed to adequately separate the maxillary and mandibular dentitions.

CBCT has also been proven to be an appropriate tool for evaluating the maxillary and mandibular bone marrow density [24,25,26] and condylar volume [27]. Thus, further exploring the feasibility of the current segmentation and superimposition protocol for assessing mandibular bone marrow density and tracking condyle modeling and remodeling is the next objective of our investigation.

No doubt, Dolphin 3D software is not inexpensive. However, we created this protocol based on Dolphin 3D because this software is not only routinely used in our clinic but is also widely utilized in graduate orthodontic programs. According to the PubMed database, an increasing number of researchers are employing Dolphin 3D software in their studies encompassing diverse areas of investigation, such as predicting the upper airway change in orthognathic patients [28], assisting in virtual surgical planning [29], evaluating dentoskeletal effects of rapid maxillary expansion [30], and even routine 2D tracing analysis [31]. Thus, we believe that our protocol is based on a widely used software, which minimizes the chances of imposing additional costs for clinicians and researchers who are likely to already own Dolphin 3D Imaging software.

Last but not least, the current study is a retrospective study using human data previously obtained for clinical treatment purposes. No additional radiographs were taken for the present study. Although technological developments have significantly reduced the radiation dosage associated with CBCT acquisition [2], we highly recommend that all clinical CBCTs taken should follow ALARA (as low as reasonably achievable) principles to avoid unnecessary radiation exposure to patients and clinicians [32].

In summary, a user-friendly 3D-imaging-based mandibular segmentation protocol is introduced in the current study, with five CBCT images from three patients selected to preliminarily demonstrate the feasibility of the protocol. Without a doubt, many more samples are needed to draw definitive conclusions in growth and development investigations and assess treatment efficacy. However, by disseminating our protocol in a timely manner, we hope that this sharing of knowledge can significantly benefit global collaborative efforts to achieve a more detailed understanding of how to better evaluate growth and development and ultimately improve one’s ability to deliver better orthodontic clinical care.

## Data Availability

The data presented in this study are contained within this article and supplementary materials.

## Supplementary Materials

The following are available online at https://www.mdpi.com/2077-0383/10/1/127/s1: Figure S1: The 3D reconstructed mandibular images of the 20 samples used for intraclass correlation coefficient analysis.
