# Supplementary figures and images for: A User-Friendly Protocol for Mandibular Segmentation of CBCT Images for Superimposition and Internal Structure Analysis
Source: J Clin Med. 2021 Jan 1;10(1):127. doi: 10.3390/jcm10010127 (PMC7796406; doi:10.3390/jcm10010127)

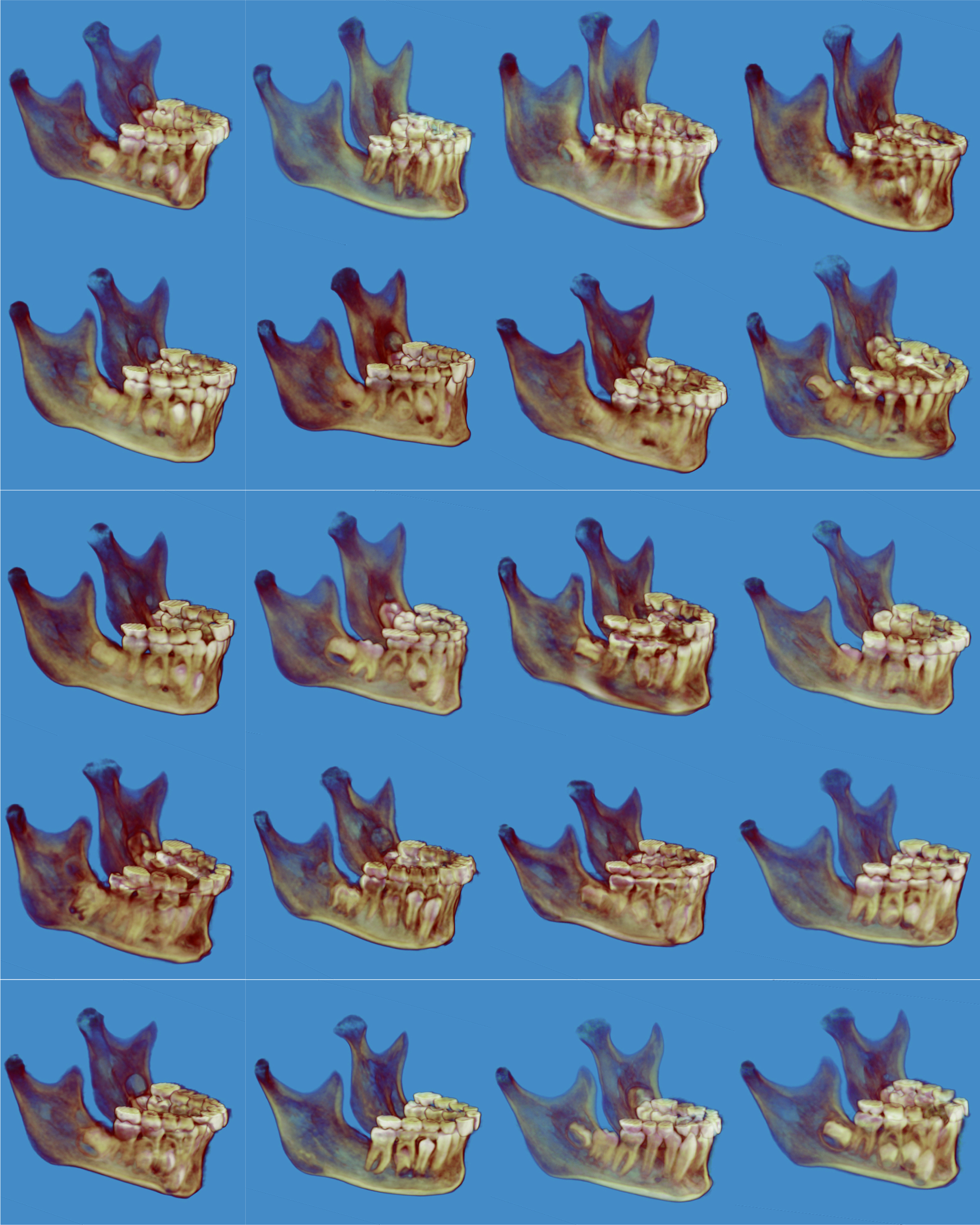

Supplement: Supplementary file 1 [file jcm-10-00127-s001.zip › jcm-1022354-supplementary.tif]
